# Engineering the Interface between Inorganic Nanoparticles and Biological Systems through Ligand Design

**DOI:** 10.3390/nano11041001

**Published:** 2021-04-13

**Authors:** Rui Huang, David C. Luther, Xianzhi Zhang, Aarohi Gupta, Samantha A. Tufts, Vincent M. Rotello

**Affiliations:** Department of Chemistry, University of Massachusetts Amherst, 710 N. Pleasant St., Amherst, MA 01003, USA; ruihuang@umass.edu (R.H.); dluther@umass.edu (D.C.L.); xianzhizhang@umass.edu (X.Z.); aarohigupta@umass.edu (A.G.); satufts@umass.edu (S.A.T.)

**Keywords:** inorganic nanoparticles, surface chemistry, peptide and proteins, monolayers, nanozyme, bioorthogonal catalysis, bacterial biofilm, tumors, stimuli-responsive, drug delivery

## Abstract

Nanoparticles (NPs) provide multipurpose platforms for a wide range of biological applications. These applications are enabled through molecular design of surface coverages, modulating NP interactions with biosystems. In this review, we highlight approaches to functionalize nanoparticles with “small” organic ligands (Mw < 1000), providing insight into how organic synthesis can be used to engineer NPs for nanobiology and nanomedicine.

## 1. Introduction

Inorganic nanoparticles can be engineered to possess useful physiochemical properties for use in applications including biomedicine and diagnostics [1,2,3,4,5,6]. While shape [7] and size [8] both play critical roles in defining nanoparticle properties, surface chemistry is likewise crucial for function and colloidal stability [9,10]. The nanoparticle surface interfaces with the external environment, and appropriately engineered surfaces can be used to regulate interactions between nanoparticles and biomolecules [11] including peptides [12], proteins [13] and nucleic acids [14].

A wide range of strategies have been used to modulate surface properties of nanoparticles (NPs), including polymer-based surface modification of nanoparticles [15,16,17,18]. These systems are quite useful; however, the intricacies of polymer structure and dynamics introduce complexities that add an additional layer to understanding and harnessing the interactions between nanoparticles and biomolecules [19]. Designing small ligand molecules to tailor the surface properties of nanoparticles provides an approach complementary to polymer coatings, providing ease of fabrication and scalability [20]. The wide range of functionalities provided by organic chemistry renders a rich toolkit, allowing for atom-by-atom control of nano–bio interactions contiguous [1]. In this review we provide an overview of approaches for controlling nanoparticle–cell and nanoparticle–protein interactions by tailoring small molecule ligands on the particle surface. Particle–protein interactions are important in their own right and also serve to modulate nanoparticle–cell interactions through corona formation. Nanoparticle interaction with nucleic acids [21] and intracellular delivery of nanoparticle-bound biomolecules [22] are likewise important related concepts that are beyond the scope of this review.

## 2. Modulating Nano–Bio Interactions through Surface Design

Engineered NPs can feature a diverse range of chemical surface functionalities to promote specific or nonspecific interaction with proteins or other biomolecules of interest. Proteins are particularly interesting targets for their roles in cellular homeostasis and metabolic diseases [23,24]. Enzymatic activity is closely linked to protein structure, creating an intriguing engineering challenge for NP design at the ligand–protein interface.

### 2.1. Regulation of Enzyme Activity

**Figure 1:**
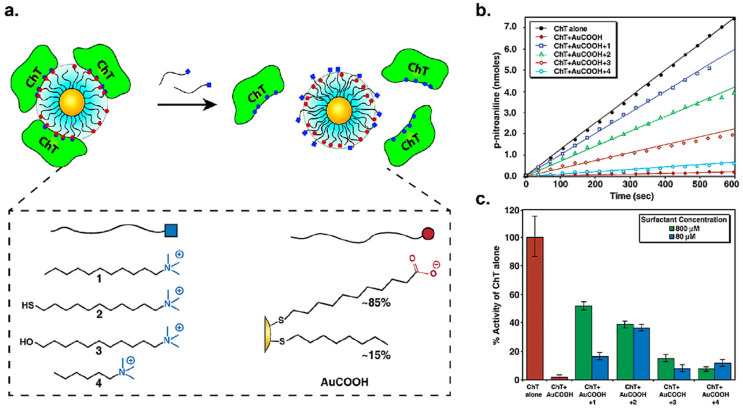
(**a**) Schematic illustration of the mechanism of rescuing chymotrypsin activity using long-chain surfactants. (**b**) Initial velocities of chymotrypsin before and after adding four different surfactants. Chymotrypsin reactivation was monitored via the hydrolysis of the chromogenic substrate *N*-succinyl-l-phenylalanine p-nitroanilide (SPNA), as measured by UV–vis spectroscopy. (**c**) Reactivation of chymotrypsin is dependent on surfactant concentration. (Adapted with permission from Reference [28]; Published by American Chemical Society, 2003).

NPs can be used as enzyme inhibitors by engineering them to noncovalently interact with protein surfaces, blocking access to the active site or triggering protein denaturation [25,26]. In early studies, the Rotello group functionalized gold nanoparticles (AuNPs) with COO-surface moieties to allow them to interact with the cationic region surrounding the active site of chymotrypsin, thereby inhibiting enzymatic activity [27]. Using these surfactant-like ligands, an almost complete denaturation of chymotrypsin was observed by circular dichroism (CD) upon addition of AuNPs. Importantly, negatively charged AuNPs showed a relatively high degree of selectivity to chymotrypsin over proteins like elastase and β-galactosidase due to electrostatic complementarity. Follow-up studies [28] demonstrated that this AuNP-based inhibition of chymotrypsin could be reversed (up to 50%) through in situ surface modification of AuNPs using long-chain surfactant (Figure 1). In related work, Hamad–Schifferli engineered a series of 9.6-nm AuNPs featuring anionic and zwitterionic functionalities to investigate the effect of surface charge on the enzymatic activity of glucose oxidase (GOx) [29]. The authors reported that neutral and zwitterionic ligands acted as a steric barrier to the active site, and further that changing AuNP surface chemistry varied binding kinetics greatly, with certain surface chemistries irreversibly affecting GOx denaturation and activity.

Rotello showed that the nature of NP–protein interaction can be tuned using CdSe NPs featuring oligo (ethylene glycol) (OEG) ligands with chain-end functionality [30]. By varying ligand composition, the authors showed tunable surface recognition of chymotrypsin, with three levels of interaction: no interaction, inhibition with denaturation, and reversible inhibition with retention of structure. This work demonstrated NPs as versatile platforms for enzyme binding and controlled inhibition.

Amino acids functionalities are attractive candidates as NP surface moieties for enzyme inhibition as they can not only provide structural diversity but also mimic naturally occurring protein–protein interactions [31]. Rotello and coworkers generated a series of L-amino acid-functionalized AuNPs with varied hydrophobicity and electrostatic charge to probe binding with the surface of chymotrypsin [32]. Determined by binding constant, the authors reported that AuNPs featuring hydrophobic groups bound more strongly than those with hydrophilic groups. Later work demonstrated that chirality plays a similarly important role in protein binding, and that unlike small molecule regulators, specific binding interactions significantly affect AuNP-based enzyme regulation [33]. 

NPs can also be functionalized with small molecule recognition elements to promote specific binding to a protein of interest for inhibition [34]. Recent work by Lira demonstrated AuNPs decorated with p-mercaptobenzoic acid ligands could bind distinct allosteric exosites of the serine protease thrombin, providing allosteric regulation of the enzyme active site without large-scale denaturation [35]. 

In contrast to enzyme inhibition, AuNPs can also be engineered to refold denatured enzymatic proteins. AuNPs bearing dicarboxylate functionalities promoted the refolding of thermally denatured cationic enzymes, including chymotrypsin, lysozyme and papain to their native conformations [36]. The Vinogradov group reported alumina NPs as agents to promote renaturation of misfolded enzymes with overall anionic charge [37]. More recently, Khare demonstrated magnetic iron oxide nanoparticles featuring aminopropyl triethoxysilane (APTES) modifications that were capable of refolding thermally denatured enzymes [38]. After incubation between APTES-NPs and thermally denatured cholesterol oxidases, dynamic light scattering (DLS), zeta potential measurements, fluorescence and CD spectroscopy confirmed enzyme refolding to the native state. Similar engineering approaches could provide an avenue for specific enzyme regulation for therapeutic purposes, including refolding of misfolded enzymes linked to diseases.

### 2.2. Modulating Interactions between NPs and Proteins

Nanoparticles administered into the blood can interact nonspecifically with serum proteins and form a shell of protein, called the protein corona [39,40,41,42,43,44]. Protein corona formation is mainly mediated by coulombic and Van der Waals forces, hydrogen bonding, and hydrophobic interactions [45]. Moreover, the wetting behavior of the NP surface also plays an important role in regulating protein adhesion behaviors [46,47,48]. Protein corona provides new “identity” to nanomaterials in the biological environment, significantly affecting NPs in terms of their biodistribution, cellular uptake and cargo release [49,50,51]. Moreover, the protein corona diminishes targeting effects of NPs and accelerates their clearance from the body through the mononuclear phagocyte system (MPS), greatly hampering therapeutic potential for nanomedicines [52]. Tuning the hydrophilicity and electroneutrality of the NP monolayer provides an effective way to reduce protein absorption on the surface [53]. 

**Figure 2:**
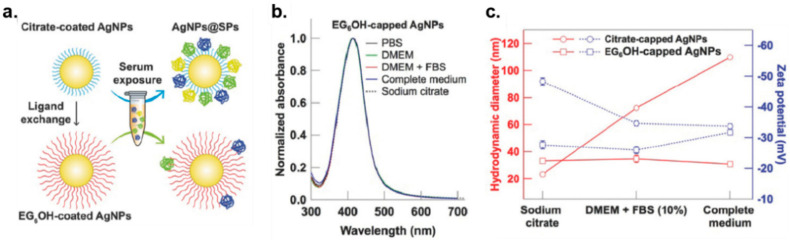
(**a**) Schematic illustration of serum protein adsorption on the surface of citrate-coated AgNPs and the formation of nonfouling AgNPs by coating OEG groups. (**b**) The stability of OEG-functionalized AgNPs was confirmed by measuring UV-vis absorption spectra in various culture medium. (**c**) Hydrodynamic diameter and zeta potential of AgNPs before and after incubation in growth media. (Adapted with permission from Reference [60]; Published by WILEY, 2018).

OEG has been widely used to functionalize NP surfaces to reduce protein corona formation [54,55,56,57]. The terminal hydroxyl group provides little to no charge to the entire system, while the ethylene glycol units provide hydrophilicity. Work by Rotello reported that AuNPs functionalized with thioalkylated OEG showed minimized protein adsorption [58]. Walker and coworkers similarly demonstrated the creation of nonfouling AuNPs using OEG groups [59]. Interestingly, rate constant of protein–AuNP dissociation was quantified by introducing nonequilibrium capillary electrophoresis of equilibrium mixtures (NECEEM) in this work. Recently, Gentili [60] reported that the use of OEG-alkanethiol on silver nanoparticles efficiently reduced corona formation in serum-containing media, as confirmed by UV–vis spectroscopy, DLS and zeta potential (Figure 2).

Incorporating zwitterionic functionalities such as amino acids onto the NP surface is an alternative to the use of OEG [61,62]. Early work by Frangioni demonstrated that functionalizing quantum dots with cysteine and tumor targeting moieties could reduce corona formation and provide “stealth” character while enhancing targeting efficiency [63]. More recently, a similar strategy was adopted to modify the surface of fluorescent silica nanoparticles (SiNPs) by Mahmoudi’s group [64]. In this study biotin was used as a ligand to target tumor cells bearing biotin receptors, with zwitterionic cysteine incorporated to avoid formation of the protein corona, as confirmed by gel electrophoresis. Studies with several cell lines, two biotin receptor-positive and another receptor-negative, demonstrated that nonfouling SiNPs have significantly improved targeting efficiency compared to counterparts modified with cysteine or biotin only. 

**Figure 3:**
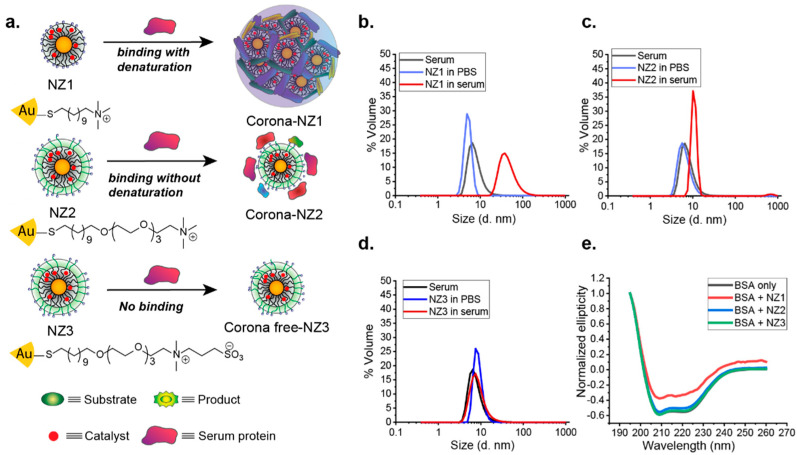
(**a**) Schematic illustration of tuning ligand monolayers to modulate interactions between AuNPs and proteins. As confirmed by dynamic light scattering (DLS) (**b**–**d**), nanozyme-2 (NZ2) formed a corona-like structure with serum proteins while NZ1 formed aggregates. Zwitterionic NZ3 showed nonfouling property in the presence of serum medium. (**e**) Circular dichroism (CD) results demonstrated that NZ1 partially denatured bovine serum albumin (BSA), inducing its conformational changes. However, NZ2 and NZ3 retained the original protein conformation. (Adapted with permission from Reference [68]; Published by American Chemical Society, 2020).

In addition to amino acids, zwitterionic betaine groups have also been widely used for functionalization to enhance NP stealth properties [65]. Rotello developed AuNPs with a series of sulfobetaine terminal groups of variable hydrophobicity, reporting no protein adsorption at physiological serum concentrations [66]. Later work by Parak and coworkers reported that quantum dots coated with sulfobetaine ligands showed negligible change in size after incubating with human serum albumin, suggesting no corona formation [67]. Recently, betaine-based zwitterionic AuNPs [68] were used by Rotello to encapsulate transition metal catalysts (TMCs) within the ligand monolayer to obtain bioorthogonal nanoezymes, or “nanozymes” (NZs) (Figure 3). Bioorthogonal NZs are artificial enzymes that can generate imaging or therapeutic agents in situ in biological systems through bond cleavage reactions [69,70,71,72]. Kinetic studies showed that NZs functionalized with zwitterionic surface functionalities maintain high catalytic activity in biological environments due to their “corona-free” properties [73,74,75].

### 2.3. Modulating NP-Cell Interactions

Cells interact with NPs mainly through Van der Waals and electrostatic interactions. These noncovalent interactions can be modulated through tuning NP physicochemical properties such as charge and hydrophobicity [76]. 

**Figure 4:**
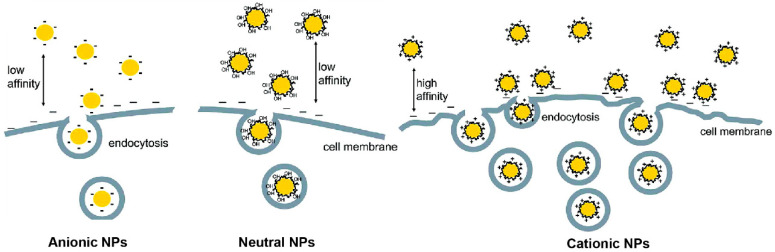
Schematic illustration of the interactions between NPs with different types of surface charges and cells. (Adapted with permission from Reference [77]; Published by American Chemical Society, 2009).

Surface charge of NPs plays an important role in their cellular uptake [77,78,79]. Typically, anionic and neutral NPs have low affinity for the anionic cell membrane whereas cationic NPs are strongly electrostatically attracted to the membrane (Figure 4).

**Figure 5:**
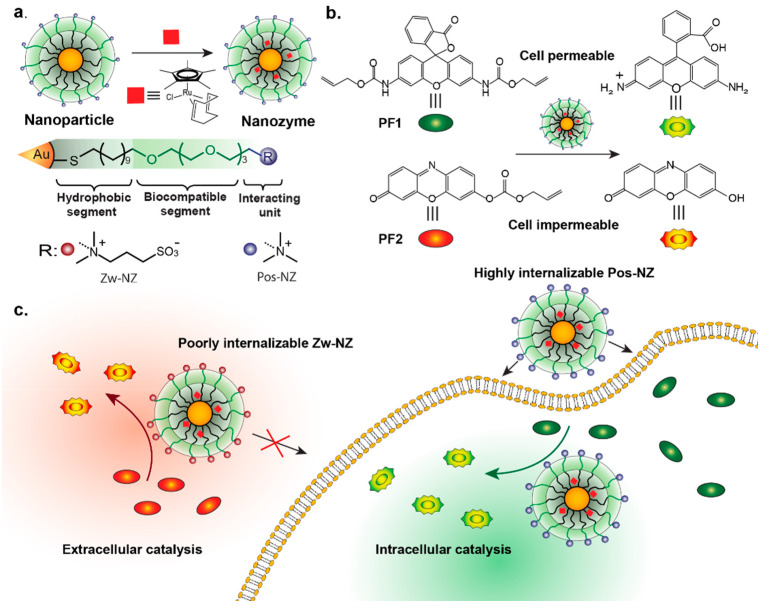
(**a**) Schematic representation of nanoparticles, nanozymes and ligand structures of zwitterionic nanozymes (Zw-NZ) and cationic nanozymes (Pos-NZ). (**b**) Structures of prodyes and their corresponding fluorescent products (rhodamine 110 and resorufin) after bond cleavage catalyzed by Ru-based complex in NZs. (**c**) Schematic representation of specific localization of nanozymes by engineering surface functionalities; activation of PF1 happens in intracellular region while that of PF2 occurs extracellularly. (Adapted with permission from Reference [75], Published by American Chemical Society, 2019).

Based on this electrostatic property, a wide range of NPs have been engineered to carry positive charge for their enhanced cellular internalization [80,81,82,83,84,85,86,87]. Additionally, surface charge modification can also be used to dictate either extra- or intracellular localization of NPs [75]. Rotello noncovalently incorporated dyes or drugs into zwitterionic AuNPs for enhanced delivery efficiency [82]. As demonstrated by fluorescence microscopy and cytotoxicity assays, encapsulated drugs or dyes were efficiently released into cells. Importantly, there is little or no cellular uptake of AuNPs due to the noninteracting nature of their surfaces with cells, as confirmed by transmission electron microscopy (TEM) and inductively coupled plasma mass spectrometry (ICP–MS). Recently, these zwitterionic AuNPs were used to encapsulate TMCs to localize the catalyst molecules specifically to the extracellular region [75]. As shown in Figure 5, the spatial control of bioorthogonal catalysis can be achieved by using positive or zwitterionic surfaces. 

NP hydrophobicity is another key parameter that affects cellular uptake [88,89]. Guével demonstrated that cationic gold nanoclusters with increasing surface hydrophobicity showed concomitantly enhanced cellular internalization [90]. Similarly, Zheng introduced hydrophobic octanethiol onto the surface of zwitterionic AuNPs to enhance their affinity for the cell membrane and observed an enhancement in cellular uptake by more than an order of magnitude [91]. 

Active targeting is a strategy to provide enhanced cellular uptake by utilizing affinity ligands on the NP surface for specific recognition with receptors overexpressed on target cells [92,93]. Small molecule ligands (e.g., folic acid, methotrexate, anisamide, cholic acid, daptomycin, fluorine and sugars) have been utilized extensively for conjugation to various inorganic NPs due to their stability, ease of modification and availability [94,95,96]. To date, several therapeutic and diagnostic approaches using active targeting NPs have entered clinical trials, with several gaining FDA approval [97].

**Figure 6:**
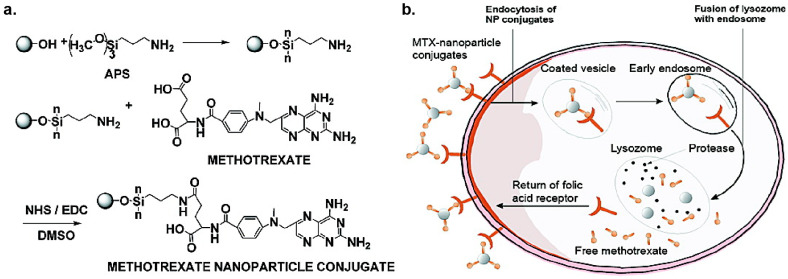
(**a**) Surface modification of magnetite nanoparticles with methotrexate (MTX). (**b**) Schematic representation of MTX release in simulated lysosomal pH conditions. (Adapted with permission from Reference [104]; Published by American Chemical Society, 2005).

Folic acid (FA) is a frequently used targeting moiety due to overexpression of the folate receptor on cancerous cells and inflamed tissue [98,99,100,101,102,103]. In work by Zhang, superparamagnetic iron oxide nanoparticles were modified with methotrexate (MTX), a small-molecule chemotherapeutic and synthetic analog of FA [104]. These NPs exhibited enhanced uptake in MCF-7 and HeLa cancer cells, with enhanced cancer cell death after MTX release triggered by low lysosomal pH (Figure 6). Similar approaches have recently seen success using AuNPs [105] and mesoporous silica [106]. Benzamides are another commonly used class of active targeting ligand for their ability to target the sigma receptors overexpressed on prostate cancer cells [107,108]. Anisamide variants have been conjugated to poly(ethyleneamine) (PEI) gold nanospheres [109] or PEGlyated AuNPs [110] to facilitate cell-specific internalization of siRNA-conjugated AuNPs, both in vitro and in vivo. Similar approaches have utilized NP conjugation of biotin [111], mannose [112,113] or hyaluronic acid (HA) [114,115] for targeted delivery to various cell types.

Chan examined the effect of diameter on tumor uptake of spherical AuNPs, with and without targeting ligand (transferrin coating). The authors reported no significant differences with particles ≤100 nm, but observed tumor accumulation five times faster and two-fold higher with targeted, transferrin-coated particles [100,116]. Later work from this laboratory critically examined tumor targeting by showing that less than 14 out of 1 million (0.0014% injected dose) intravenously administrated targeted NPs were delivered to targeted cancer cells, with the majority trapped in the extracellular matrix or phagocytosed by macrophages [117]. This work helped to show that a re-examining of the active targeting process is necessary for translation of NP therapeutics, and that interaction between NPs and cells is significantly more complicated when moved in vivo.

## 3. Modulating Nano-Bio Interactions through Stimuli-Responsive NPs

NP localization at both the organismal and cell level remains a challenge to nanotherapeutics. Engineering NPs for stimuli responsiveness is an effective strategy to enhance NP localization at a desired locale [118,119]. Versatile NP ligands can be designed to respond to optical stimuli, pH differences and enzyme-induced cleavage.

### 3.1. Enzyme-Induced Aggregate Formation

**Figure 7:**
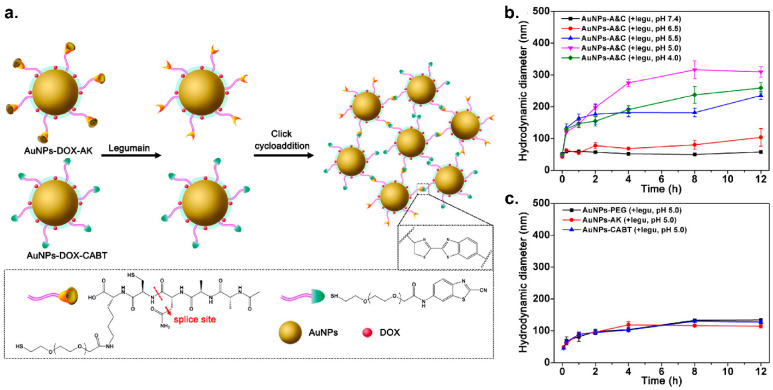
(**a**) Schematic representation of the legumain-triggered AuNP aggregation and composition of AuNPs functionalized with peptides (Ala-Ala-Asn-Cys-Lys) (AuNPs-AK) and AuNPs grafted with 2-cyano-6-amino-benzothiazole (AuNPs-CABT). (**b**) The proteolytic capacity of legumain is pH dependent. As a result, AuNP aggregation triggered by legumain varied at different pH values and the size increase was the highest at pH 5.0, as confirmed by DLS measurement. (**c**) AuNPs functionalized with PEG, AuNPs-AK only or AuNPs-CABT only was used as controls. DLS data showed that no aggregation was observed when treated with legumain at pH 5.0. (Adapted with permission from Reference [122], Published by American Chemical Society, 2016).

Efficient cellular uptake is crucial for nanoparticle use in bioapplications, including diagnosis and therapy [77,88]. Stimuli-responsive aggregation has been widely used as a way to enhance accumulation of NPs in cells [120]. Once AuNPs aggregate inside cells, exocytosis will be blocked and their backflow to the bloodstream will be restricted, effectively enhancing cellular retention of AuNPs [121]. Gao created a nanoscale platform consisting of AuNPs functionalized with peptides (Ala-Ala-Asn-Cys-Lys) (AuNPs-AK) and AuNPs grafted with 2-cyano-6-amino-benzothiazole (AuNPs-CABT) to trigger intracellular aggregation [122]. With this system, peptide-modified AuNPs undergo ligand hydrolysis in the presence of the proteolytic enzyme legumain, which triggers the formation of aggregates due to a click cycloaddition reaction between the newly formed 1,2-thiolamino moiety and the contiguous cyano group (Figure 7). Later studies in murine glioma models showed that this AuNP-based nanoplatform was able to aggregate rapidly upon entering into glioma cells, resulting in an increased amount of AuNPs internalized and greater tumor cell death when compared to nonaggregated counterparts. Later work from this group introduced new elements to the AuNP ligands to improve their membrane permeability and target different tumor models. Octaarginine and arginylglycylaspartic acid [123] were simultaneously conjugated to the ligand for an enhanced AuNP accumulation in glioblastoma cells while cediranib [124] was grafted for that in 4T1 cells.

### 3.2. pH-Dependent Aggregate Formation

**Figure 8:**
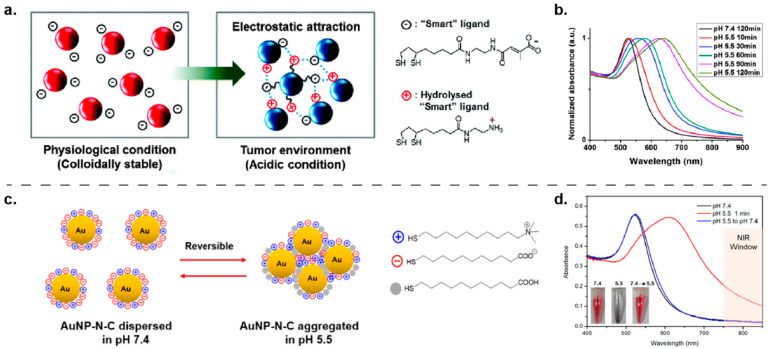
Schematic representation of pH-triggered AuNP aggregation through two different mechanisms. (**a**) The surface charge of AuNPs is designed to switch from negative to a mixture of negative and positive charges through bond cleavage under the mildly acidic conditions; once the surface charge is neutralized, AuNPs begins to aggregate due to the absence of electrostatic repulsion. (Adapted with permission from Reference [130]; Published by Royal Society of Chemistry, 2016). This transformation was monitored by (**b**) UV–vis spectroscopy for 2 h. (**c**) The surface charge of AuNPs is designed to be neutralized owing to the protonation of MUA ligand under the mildly acidic conditions. As demonstrated by UV–vis spectroscopy (**d**), this transformation is reversible. (Adapted with permission from Reference [132]; Published by American Chemical Society, 2017).

Another common stimulus to trigger nanoparticle aggregation is pH, a property can be used to target acidic microenvironments such as those found in tumors or bacterial biofilms [125,126,127,128]. Early work by Kim demonstrated that nanoparticles can respond to pH changes and form aggregates [129]. Hydrolysis-susceptible citraconic amide was used to functionalize AuNPs for its ability to undergo partial bond cleavage at a pH lower than 7, forming positively charged primary amines. Upon hydrolysis, the surface charge is neutralized and AuNPs begins to aggregate in the absence of electrostatic repulsion. Compared to counterparts with permanent negative charge, pH-responsive AuNPs demonstrated enhanced cellular uptake in B16F10 and NIH 3T3 cells, confirmed by dark field optical microscopy. This platform was later used for photoacoustic imaging [130] and showed a cancer-specific AuNP accumulation at the cellular level (Figure 8a). Inspired by the Kim group, Wong and coworkers functionalized AuNPs with a hydrolysis-susceptible citraconic amide moiety for pH-responsiveness and a peptide for active tumor-targeting ability [131]. Engineered AuNPs demonstrated efficient cellular uptake in U-87MG cells both in vitro and in vivo.

In addition to pH-induced bond cleavage, pH-dependent protonation/deprotonation transition has also been used as a strategy for surface neutralization to trigger aggregation of nanoparticles. Grzybowski and coworkers developed a series of zwitterionic AuNPs using both *N,N,N*-trimethyl(11-mercaptoundecyl)ammonium ion (TMA) and 11-mercaptoundecanoic acid (MUA) as ligands for surface modification [133]. The protonation of the MUA ligand at low pH can neutralize the surface charge of AuNPs to form aggregates through Van der Waals attractions and hydrogen bonding. Importantly, the precipitating pH value of these zwitterionic AuNPs can be engineered by tuning MUA/TMA ratio, providing an avenue for pH-specific biological targeting. Using a similar approach, Ji fabricated a series of pH-responsive zwitterionic AuNPs for tumor treatment [134]. It was observed that optimized AuNPs were stable at the pH of blood and normal tissues but aggregated rapidly in response to acidic tumor environment, rendering a significantly enhanced cellular internalization of particles compared to nonsensitive PEGylated AuNP controls. More recently, this platform was used to treat methicillin-resistant *Staphylococcus aureus* (MRSA) bacterial biofilm (Figure 8c). In animal studies, the aggregation of AuNPs triggered by acidic biofilm microenvironment facilitated localization in the biofilm extracellular polymeric substance (EPS), allowing for effective NIR photochemical therapy with minimal toxicity to normal healthy cells [132]. To further develop this system, Li performed coarse-grained (CG) molecular dynamics simulations to systematically investigate the stability of MUA/TMA-based pH-responsive AuNPs and their interactions with cells [135]. 

### 3.3. pH-Responsive Charge Conversion

As mentioned previously, cationic nanoparticles generally display significantly higher cellular internalization compared to zwitterionic counterparts due to electrostatic attraction with the negatively charged cell membrane [78,79]. Based on this property, the Rotello group functionalized AuNPs with a pH-responsive sulfonamide-based ligand of which the surface charge can switch from zwitterionic to cationic at mildly acidic conditions to enhance cellular uptake in the acidic tumor microenvironment [136]. ICP–MS data showed that cellular uptake of AuNPs at pH 6.0 was four-fold higher than that at pH 7.4 and no aggregation was observed under any conditions, as confirmed by DLS. Recently, the You group utilized a similar sulfonamide-based ligand to modify the surface of their gold nanocages [137]. After intravenous injection into 4T1 murine breast tumor models, pH-responsive AuNPs exhibited efficient accumulation within tumor cells. 

**Figure 9:**
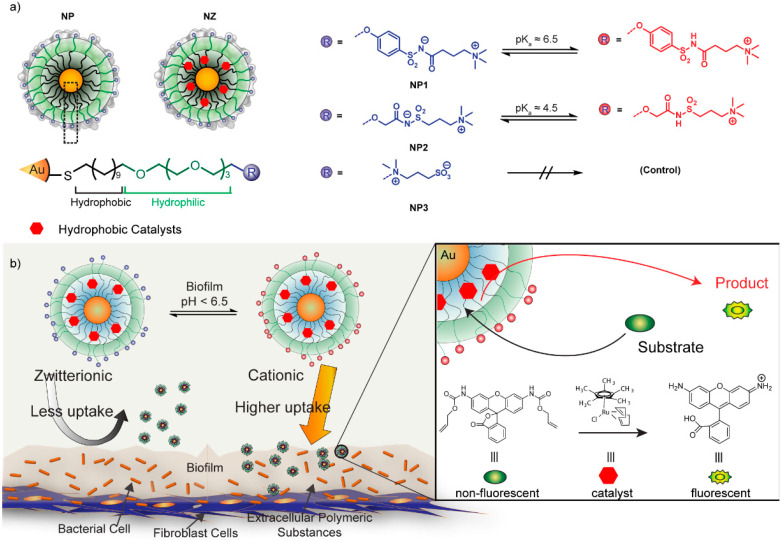
(**a**) Schematic representation of nanoparticles, nanozymes and molecular structures of pH-switchable and control ligands on AuNPs. (**b**) Schematic representation showing selective targeting of biofilm infections using pH-responsive nanoparticles and NZ-mediated fluorogenesis of prodye inside of biofilms. (Adapted with permission from Reference [138]; Published by American Chemical Society, 2018).

Similar approaches have been taken to target the acidic microenvironment of bacterial biofilm infections. In 2018, Rotello group investigated the interaction of pH-responsive AuNPs featuring sulfonamide moieties with bacterial biofilms [138]. Zwitterionic AuNPs transitioned to cationic at the acidic biofilm pH, essentially “honing” to the infection due to strong electrostatic interaction (Figure 9). In a fibroblast–biofilm coculture model, AuNPs selectively penetrated and accumulated inside biofilms. In an alternative approach, carboxyl betaines were recently used by Luo to modify the surface of silver nanoparticles, resulting in enhanced adhesion of nanoparticles to the bacteria membrane [139].

## 4. Conclusions

As demonstrated in this review, engineered NP surface chemistry provides a powerful avenue to tailor the physicochemical properties of NPs, offering potential to surpass obstacles and enhance efficiency in nanomedicine. The examples highlighted herein demonstrate the power of NP surface functionalization to control nano–bio interactions, from modulation of enzymatic activity to selective localization in specific cell types. Nanomaterials undergo complex interactions with biomolecules and cell surfaces, and the chemical versatility granted by NP ligands thus remains an unmatched tool in exploring the nano–bio interface.

Organic chemistry provides an immense range of chemical diversity for the functionalization of NPs. The breadth of chemical functionalities available provides a rich toolkit to probe nano–bio interactions. Future research will continue to move toward precise control over biochemical interactions and provide a deeper understanding of structure–function relationships. Such studies will further extend the potential of NP platforms in therapeutics, imaging and diagnostics.
